# Host exposure history modulates the within-host advantage of virulence in a songbird-bacterium system

**DOI:** 10.1038/s41598-019-56540-6

**Published:** 2019-12-30

**Authors:** Ariel E. Leon, Arietta E. Fleming-Davies, Dana M. Hawley

**Affiliations:** 1 Department of Biological Sciences, Virginia Tech, Blacksburg, VA 24061 USA; 2 Biology Department, University of San Diego, San Diego, CA 92110 USA

**Keywords:** Evolutionary ecology, Bacterial infection

## Abstract

The host immune response can exert strong selective pressure on pathogen virulence, particularly when host protection against reinfection is incomplete. Since emerging in house finch populations, the bacterial pathogen *Mycoplasma gallisepticum* (MG) has been increasing in virulence. Repeated exposure to low-doses of MG, a proxy for what birds likely experience while foraging, provides significant but incomplete protection against reinfection. Here we sought to determine if the within-host, pathogen load advantage of high virulence is mediated by the degree of prior pathogen exposure, and thus the extent of immune memory. We created variation in host immunity by experimentally inoculating wild-caught, MG-naïve house finches with varying doses and number of exposures of a single pathogen strain of intermediate virulence. Following recovery from priming exposures, individuals were challenged with one of three MG strains of distinct virulence. We found that the quantitative pathogen load advantage of high virulence was strongly mediated by the degree of prior exposure. The greatest within-host load advantage of virulence was seen in hosts given low-dose priming exposures, akin to what many house finches likely experience while foraging. Our results show that incomplete host immunity produced by low-level prior exposure can create a within-host environment that favors more virulent pathogens.

## Introduction

Pathogens demonstrate extensive variation in the amount of harm, or virulence, they cause their hosts. For directly-transmitted pathogens, high virulence presents a paradox because the increased morbidity and mortality that virulent pathogens cause their hosts should reduce the likelihood of their successful transmission by reducing opportunities for contacts with susceptible hosts. The “trade-off” hypothesis helps to resolve this paradox by considering virulence as an unavoidable consequence of the within-host pathogen replication necessary for ongoing transmission^1,2^. Thus, any factors that alter the ability of a pathogen to replicate within a host can cause strong selection on pathogen virulence^3,4^.

There is growing interest in understanding how the within-host environment modulates selection for virulence [e.g.^3,5–8^]. The immune response of a host has been shown to select for more virulent pathogens in cases where the host forms specific immunity against a pathogen, whether via vaccination, previous exposure, or innate immune priming^3,9–12^. If a host has incomplete immune protection, meaning they are able to be reinfected by the same type of pathogen, this provides a particularly strong selective environment for virulence^3,6,11–14^. As predicted by theory, virulent strains often have higher within-host replication rates^15,16^, and thus may be more likely to replicate at levels needed to overcome incomplete host immunity and successfully colonize incompletely-immune hosts. Additionally, when incomplete immunity protects hosts from infection-induced morbidity and mortality, there are fewer evolutionary costs for a pathogen to maintain high levels of virulence^7^. Thus, virulent pathogens should have an increased likelihood of competing and persisting in a population with a high prevalence of incompletely-immune hosts^3,11^.

The degree of immunity that a host harbors is strongly mediated by the extent of prior exposure to a pathogen (i.e., dose, number of exposures) that an individual receives^17^. Thus, the strength of within-host selection for virulence from existing host immune responses is likely to be mediated by the exposure level that a host initially receives, and the amount of immunity subsequently produced. While past studies have examined how imperfect vaccination^18^ or immunity generated by high dose exposures^11^ select for more virulent pathogens, the role of initial exposure level in driving selection for pathogen virulence has not yet been examined. In natural settings, hosts are often exposed to varying levels of pathogen, and within a population, there may be significant heterogeneity in the exposure dose and total number of exposures to a given pathogen [e.g.^19^]. Low-level exposure has been found to produce immunity to a homologous pathogen strain akin to that produced by imperfect vaccination [e.g.^17,20^], suggesting that this type of exposure may play a significant role in driving virulence evolution in natural populations.

Here we use a natural host-pathogen system where virulence has been shown to be increasing^21,22^ to determine the role of prior exposure level in producing incomplete immunity and altering the within-host environment to favor strains of higher virulence. Free living house finch populations in North America experience seasonal epidemics of conjunctivitis caused by the bacterial pathogen *Mycoplasma gallisepticum* (MG)^23^. Since its emergence in 1994, MG has been found to be increasing in virulence in two geographically isolated populations^22^. This system meets the assumptions of the classic trade-off model, with more virulent strains leading to higher within-host exploitation^22^ and among-host transmission^21,24^. It thus provides a rare opportunity to study the potential ecological factors underlying virulence evolution in an unmanaged, free-living species. Recent work from Fleming-Davies *et al*.^11^ provided both empirical and theoretical evidence that incomplete immunity formed from prior exposure to MG can favor the evolution of increased virulence in this system. However, the immunity assessed in Fleming-Davies *et al*. (2018) was produced from single high dose exposures to MG, which are likely not representative of the range of exposure types experienced in free-living populations. MG is primarily transmitted at feeders while animals forage^25,26^, and the pathogen degrades quickly in the environment^25^. Thus, it is likely that wild birds are exposed to heterogeneous amounts of the pathogen. In addition, wild house finches have been shown to recover from natural infections at rates of over 50%, producing a pool of recovered individuals potentially susceptible to reinfection^27^. In our previous work, we found that repeated low-level exposure to MG in house finches, a proxy for what birds may experience at feeders in the wild, produces largely incomplete immune protection against secondary high-dose challenge^17^. Further, host protection against secondary infection becomes progressively more complete with higher degrees of prior host exposure^17^. Thus, the degree to which host immunity favors virulent pathogen strains in this system is likely to vary with the extent of prior host exposure to the pathogen, and the extent of incomplete immunity.

In order to test if the level (dose and number of exposures) of prior exposure mediates the quantitative pathogen load advantage of virulence in this system, we created variation in prior exposure by experimentally inoculating wild-caught, MG-naïve house finches with varying doses and number of exposures of a single pathogen strain of intermediate virulence (VA1994). Following recovery, we assessed quantitative pathogen loads of three MG strains previously shown to have distinct virulence levels^11,22^ in hosts with varying degrees of immunity generated from prior exposure. We predicted that, as previously shown^11^, the most virulent strain would maintain the highest within-host pathogen load, regardless of host prior exposure. However, we predicted that the level of prior exposure, and thus the degree of pre-existing immunity in hosts, would mediate the extent of the quantitative pathogen load advantage for virulent strains. Specifically, we predicted that virulent strains would have the greatest advantage in individuals that previously received low-level exposure, which generates largely incomplete immunity, as compared to the more complete immunity produced from previous high dose exposure. Finally, we predicted that incomplete immunity would lower the costs of virulence to the pathogen by reducing the degree of infection-induced morbidity caused by virulent strains.

## Methods

### Design and timeline

**Figure 1:**
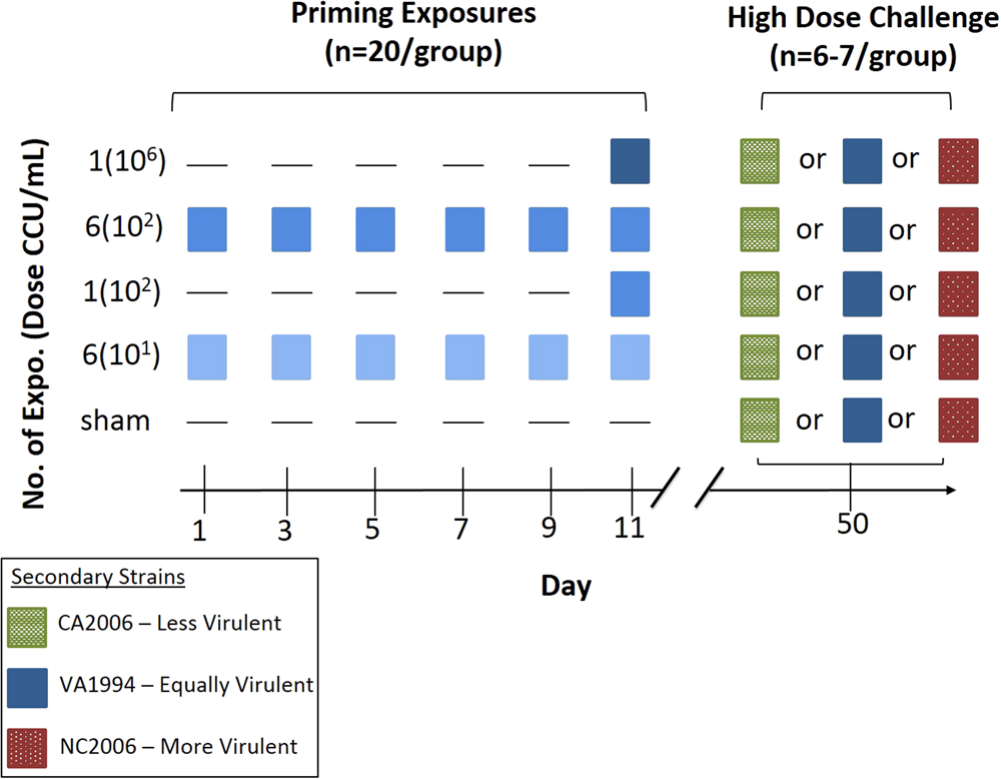
Experimental design and timeline. Individuals were given one of five priming exposure treatments that varied by dose and total number (y-axis). The strain used for priming exposures, a medium virulence strain (VA1994), is seen in blue, where the gradient in color indicates variation in dose (color intensifies with increasing dose). After recovery from priming exposures, all priming treatment groups, including the sham priming group, were randomly divided three ways and challenged with a high dose of one of three strains varying in virulence. Secondary strains included a less virulent strain (CA2006, seen in green), an equally virulent strain homologous to priming exposures (VA1994, seen in blue), and a more virulent strain (NC2006, seen in red).

To determine if low-dose and repeat exposures produce a within-host environment which favors more virulent strains, house finches were given variable priming exposures to generate variation in the degree of pre-existing immunity, and then challenged with one of three MG strains known to vary in virulence (Fig. 1; Table S1). For priming exposures, 104 wild-caught birds determined to be naïve to MG (see below) were given 1 or 6 priming inoculations that varied in dose (10^1^, 10^2^, or 10^6^ color changing units per ml [CCU/mL] of MG or a sham treatment of media alone). All priming inoculations, with the exception of sham controls, were done with a medium virulence strain of MG^17^. Repeated priming exposures were given every other day, to allow the same recovery window across treatments (Fig. 1). Priming exposures were selected based on previous work, which demonstrated that repeated exposure to 10^2^ CCU/mL resulted in a higher proportion of incomplete protection relative to a single, high dose exposure (10^6^)^17^. However, because repeated exposure to 10^2^ CCU/mL produced infection and disease in 100% of individuals and thus may not represent a lower threshold of infective dose in the wild, here we additionally incorporated a dose one order of magnitude lower (6 exposures to 10^1^). For clarity, priming treatments will be referred to using the following format: [number of exposures (priming dose)]. As an example, [6(10^1^)] represents six exposures to a dose of 10^1^ CCU/mL.

Thirty-nine days after the final priming exposure, a recovery window consistent with our previous low-dose priming experiment^17^ and designed to fall within the window of seasonal epidemics^28^, birds were challenged with a high dose of one of three strains ranging in virulence. Birds were either given a less virulent, equally virulent (homologous strain), or more virulent strain to test the quantitative pathogen load of strains of varying virulence in previously exposed hosts (Fig. 1). Sex ratios were even in priming treatments, but because many secondary group had a sample size of seven (Table S1), groups were randomly assigned a 3:4 or 4:3 male:female ratio. Final sample sizes in some treatments were reduced due to chronic infection from primary treatment (n = 1) or unanticipated mortality (n = 6), which was independent of experimental treatments (see Supplemental Materials).

### Bird capture and housing

Hatch-year house finches (n = 104) were captured in Montgomery County and Radford City, VA in June-August 2015 using a combination of mesh wire traps and mist-nets under permits from VDGIF (050352) and USFWS (MB158404-1). After capture, animals underwent a two-week quarantine protocol where they were pair-housed, monitored for visible eye lesions and bled on day 14 post-capture to test for MG-specific antibodies [as per^29^]. Only individuals that were seronegative, did not show clinical signs and had never been housed with an infected individual were included in the experiment. All finches were held at constant day length and temperature and fed an *ad libitum* diet (Daily Maintenance Diet, Roudybush Inc. Woodland, CA) prior to and during the experiment. Two weeks before the start of the experiment, birds were moved to individual housing, and remained single-housed throughout the study. All protocols for animal capture, housing and procedures were approved by Virginia Tech’s Institutional Animal Care and Use Committee and performed in accordance with Public Health Service (PHS) Policy on Humane Care and Use of Laboratory Animals.

### Pathogen strains and inoculations

We used three strains shown in prior work to vary in both the degree of within-host exploitation^11,22^, as well as the severity of clinical disease produced during infection, which predicts mortality risk in the wild^27,30^. All priming inoculations were given with a medium virulence strain “VA1994” (7994-1 7 P 2/12/09)^31^. Priming inoculation dilutions were calculated using the starting viable count of 2.24 × 10^7^ CCU/ml of VA1994. Our results in comparison to prior work^17^ which used only the homologous strain for both priming and challenge inoculations suggest that the priming doses given here were slightly higher than those used in prior work, likely due to differences in the inoculum stock. Thus, equivalent priming treatments (i.e., [1 (10^2^)]) were likely of higher actual dose and resulted in stronger protection in this study than in our prior work.

Secondary inoculations were given using three strains known to vary in virulence^11,22^; “CA2006” (2006.052-5 (4P) 1/24/10)) was used as a less virulent strain, VA1994 was used as an equally virulent homologous strain, and “NC2006” (2006.080-5 (4P) 7/26/12) was used as a more virulent strain. Previous work supports a small increase in protection due to homology of strains used across inoculations^11^. However, in that same study, the effect of virulence of the priming strain was much stronger than the increased protection from homology^11^, and thus we did not expect strain homology to alter the effects of interest. Additionally, while these strains vary in virulence, they have very low levels of genomic^32^ and antigenic variability^33^. All inocula were provided by D.H. Ley, North Carolina State University, College of Veterinary Medicine, Raleigh, NC, USA. For more information regarding inocula and inoculations, see Supplementary Materials.

### Quantitative pathogen load and disease severity

Quantitative pathogen load was measured in conjunctival tissue using quantitative PCR (qPCR), with DNA extracted from swabs of both conjunctivae (see Supplemental Materials for methods)^22,34^. Disease severity following secondary challenge was used a proxy for the costs of virulence experienced by pathogen strains in hosts of varying immune backgrounds. Disease severity directly predicts capture time in mock-predation events (i.e., a human capturing the bird by hand) in captivity^30^ and the presence of conjunctivitis predicts finch mortality in the wild^27^, and is thus a reasonable proxy for host mortality and the costs associated with pathogen virulence. Disease severity was quantified by scoring clinical signs of conjunctivitis on a scale of 0-3 per eye. Scores vary based on the degree of visible conjunctival inflammation, eversion and exudate^35^. Clinical scores (“eye scores”) were recorded for each sampling point and summed between eyes for a given sampling date. All scoring was done blind to treatment.

Animals were sampled for pathogen load and the severity of clinical signs on post-secondary inoculation days 4, 7, 14, 21 and 28. However, only a subset of pathogen load samples that captured the relevant temporal dynamics of infection were analyzed (days 7, 14, 21 post-secondary exposure). Additionally, animals were sampled for quantitative pathogen load and clinical signs of infection on pre-inoculation day -15 to ensure that all individuals included in the experiment were not harboring pathogen prior to the study, as well as on post-secondary inoculation day 0 to ensure all animals had cleared infection prior to secondary inoculation. Because responses to priming exposure levels were previously examined^17^ and our main objective was to assess traits key to pathogen fitness during secondary exposure, we have not included primary infection sampling methods here (see Supplementary Materials).

### Analysis

All analyses were done using the statistical software R^36^. Quantitative pathogen load and disease severity, measured as scorable eye lesions, were analyzed separately. Although these metrics positively covary^22^, they represent distinct biological responses that have been found to vary separately in response to exposure level^17^. For information on primary infection analyses and results, see Supplemental Materials.

#### Quantitative pathogen load

Pathogen loads from secondary infection timepoints 7, 14 and 21 from each individual were analyzed using a linear mixed model to account for repeated measures [R package lme4^37^]. Fixed effects included priming treatment, secondary strain, and the interaction between priming treatment and secondary strain, which was our main interaction of interest in this study. Individual ID was included as a random effect. All explanatory variables were treated as categorical. Additional models were run, one excluding control (sham) animals, to assess to what extent effects were driven by priming dose per se, and another including homology (yes/no) to quantify the effect of strain homology on secondary quantitative pathogen loads. Sidak post-hoc pair-wise comparisons were done among priming treatment groups within each secondary strain [R package lsmeans^38^].

Variable level effects of each categorical variable were determined using Type III Wald tests where significant interactions were detected, otherwise Type II tests were used [R package car^39^]. Additionally, based on the patterns of variation seen across priming exposure levels and secondary strains during secondary infection, we calculated the coefficient of variation (CV) for the mean of each group. Since these data were lognormally distributed, CV was calculated using the following formula for a lognormal distribution:

$$CV=\sqrt{{e}^{{s}^{2}}-1}$$

where *s* is the sample standard deviation of the sample mean for our log transformed pathogen load data^40^. The 95% confidence intervals for CV were calculated using a nonparametric bootstrap to resample the data 10,000 times in R.

#### Disease severity

Disease severity was measured using scorable eye lesions, and values from post-secondary inoculation days 7, 14 and 21 were included from each individual. The raw eye score values were natural log (ln) transformed before analysis to meet the assumption of normally-distributed data. Log transformed eye scores were then analyzed using a linear mixed model^37^. As in the pathogen load model, fixed effects included priming treatment, secondary strain, and the interaction between priming treatment and secondary strain. A random effect of individual ID was included to account for repeated measures. Effects test were conducted using the same methods listed above.

## Results

### Quantitative pathogen load

**Figure 2:**
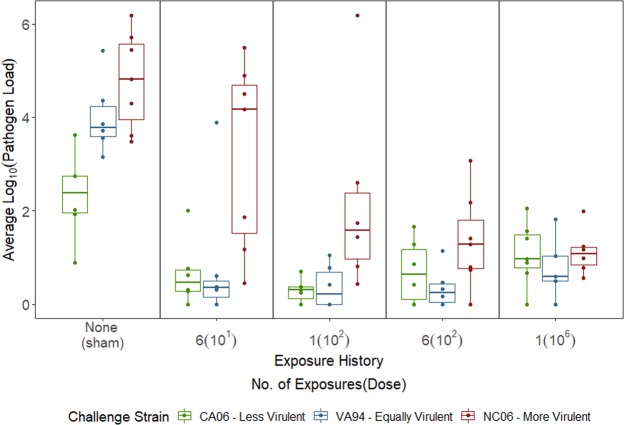
Quantitative pathogen loads following a secondary high-dose challenge with one of three strains of *Mycoplasma gallisepticum* which vary in virulence. Relative to sham priming treatments, quantitative pathogen loads decreased with any prior host exposure to pathogen (x-axis). The most virulent strain (red) had the highest quantitative pathogen loads overall, but this was mediated by priming exposure level (x-axis). Average pathogen load includes conjunctival loads averaged across secondary infection sampling time-points. Each point represents an individual.

The quantitative pathogen load advantage of strain virulence was significantly mediated by host immune background (Fig. 2). Specifically, pathogen load was significantly influenced by the virulence of the challenge strain (secondary strain), the immune background of the host (priming treatment), and their interaction (individuals = 95, observations = 284; priming exposure: LR = 13.50, df = 4, *P* = 0.009; secondary strain: LR = 17.81, df = 2, *P* = 0.0001; priming exposure * secondary strain: LR = 19.89, df = 8, *P* = 0.01; Fig. 2; Table S4). Consistent with prior work^17,22^, quantitative pathogen load increased with strain virulence and decreased with priming exposure treatment (Fig. 2; Table S4), but the nature of this decrease varied across strains, leading to the statistical interaction between strain virulence and priming exposure treatment. For the most virulent strain, pathogen loads decreased in a linear pattern with increasing priming exposure level (Fig. 2; Table S5). In contrast, for the low and mid-virulence strains, all priming exposures resulted in significantly equivalent levels of protection against secondary challenge, and thus protection from priming was largely qualitative regardless of dose (Table S5). This discrepancy in the ability of strains to overcome variable degrees of immunity from prior exposure resulted in the strongest quantitative pathogen load advantage of virulence in hosts that experienced low-level, repeated priming exposures (Fig. 2). In the 6[10^1^] treatment group, the most virulent strain was able to maintain relatively high within-host pathogen loads relative to sham controls, whereas hosts in this treatment group showed almost complete protection from mid- and low-virulence strains (Fig. 2).

The effect of challenge strain virulence (secondary treatment) and its interaction with priming treatment remained qualitatively similar when the sham control group was excluded from the analysis (secondary strain: LR = 23.55, df = 2, *P* < 0.0001; priming exposure * secondary strain: LR = 11.92, df = 6, *P* = 0.063; Table S6), and consistent with prior work^11^, results were robust to the addition of homology effects to the model (Table S7). However, when the sham control group was excluded, the main effect of priming treatment was lost (individuals = 76; observations = 227; priming exposure: LR = 1.83, df = 34, *P* = 0.61; Table S6), suggesting this effect was primarily driven by the differences between control animals and primed individuals.

**Figure 3:**
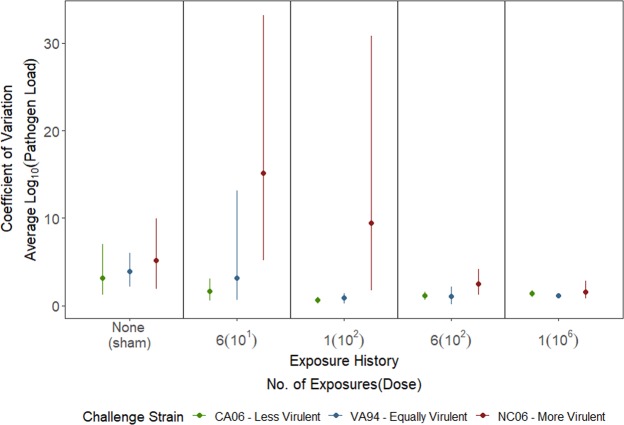
The coefficient of variation (CV) calculated for quantitative pathogen loads of house finches in response to a secondary high-dose challenge with one of three strains of Mycoplasma gallisepticum that vary in virulence. Variation was highest for the most virulent strain (red), with the highest levels of variation seen in finches with previous low-level exposure (x-axis). Points represent CV calculated using the mean for each group; 95% confidence intervals were calculated using a nonparametric bootstrap.

Interestingly, within-group variation in pathogen load, assessed with the coefficient of variation (CV), was not uniform across strains or host immune background (Fig. 3). The most virulent strain had the greatest amount of variation in quantitative pathogen loads, particularly for individuals with previous low-level exposure (Fig. 3).

### Disease severity

**Figure 4:**
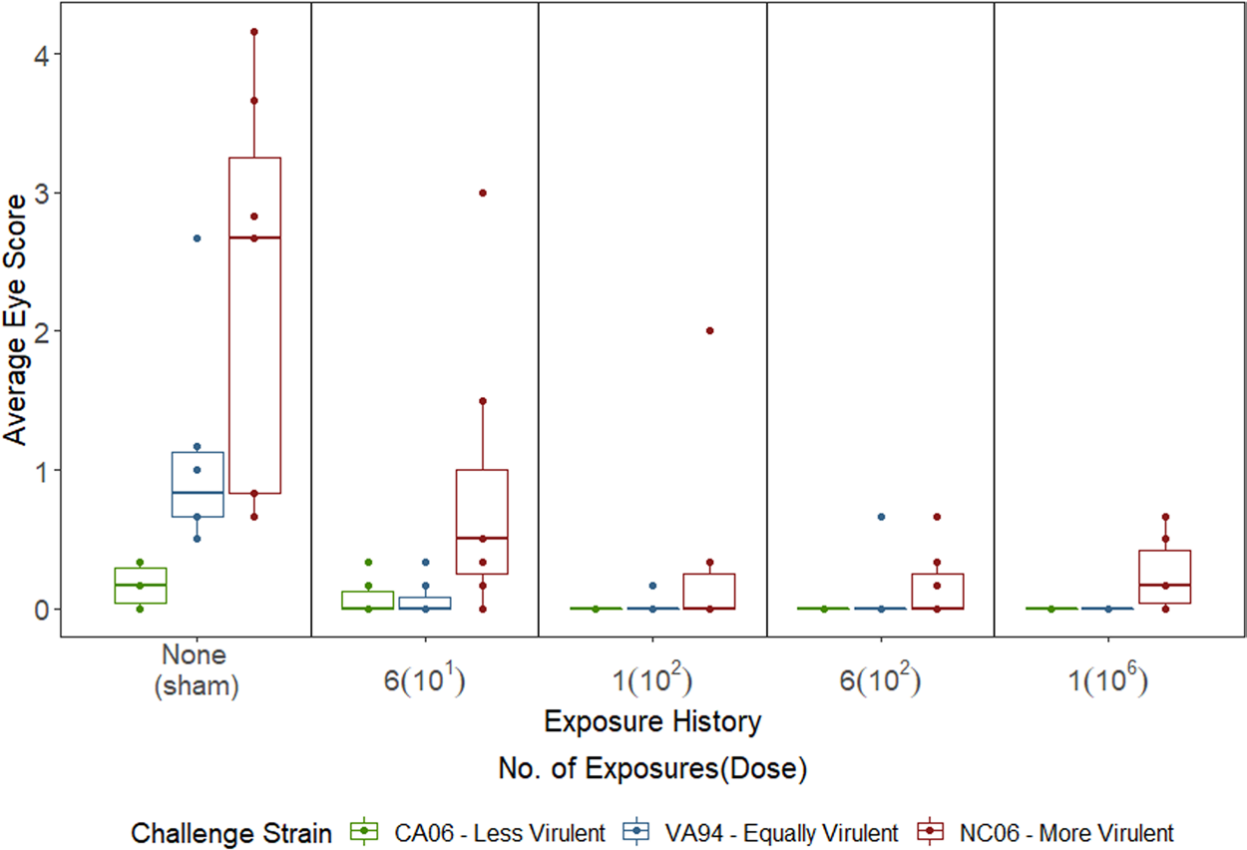
Disease severity in house finches in response to a secondary high-dose challenge with one of three strains of Mycoplasma gallisepticum which vary in virulence. Relative to sham priming treatments, eye scores were strongly reduced by any prior exposure to MG, but the extent of the reduction due to prior exposure varied across strains (x-axis). The most virulent strain (red) produced the highest overall severity regardless of priming exposure level. Eye scores were measured on a scale of 0–3 for each eye and summed across eyes. Averaged eye scores include scores for both eyes summed across secondary infection sampling time-points. Each point represents an individual.

Disease severity was also significantly influenced by the virulence of the secondary challenge strain and its interaction with priming exposure level (individuals = 95, observations = 284; secondary strain: LR = 46.53, df = 2, *P* = <0.0001; priming exposure * secondary strain: LR = 26.47, df = 8, *P* < 0.001; Fig. 4; Table S8). Overall, disease severity increased with strain virulence, but this effect was mediated by host immune background (Fig. 4; Table S8). Similar to the results for quantitative pathogen load, increased priming exposure level greatly reduced disease severity for the most virulent strain, but for the less virulent and equally virulent strains, any previous exposure to MG produced similar levels of protection from disease (Fig. 4). There was not a significant overall effect of priming treatment alone on disease severity (priming exposure: LR = 1.38, df = 4, *P* = 0.84).

## Discussion

By experimentally manipulating host prior exposure level of house finches to MG, and thus creating variation in pre-existing immunity^17^, we showed that the within-host pathogen load advantage of virulent pathogens is mediated by prior exposure level. Our results provide evidence that incomplete host immunity produced by low-dose repeat exposure creates a within-host environment which favors more virulent pathogens. Together with prior work^11^, these results suggest that incomplete immune protection formed during natural infections will likely favor the evolution of more virulent pathogens in populations where re-infections are common.

The primary objective of this study was to experimentally determine if the strength of within-host advantage for virulent strains is mediated by the degree of prior pathogen exposure, and thus the extent of pre-existing immune protection. Prior work^17^ showed that low-level exposure of house finches to MG, which many birds likely experience at feeders while foraging^25,41^, generates detectable but largely incomplete protection in house finches. This is theorized to select for more virulent pathogens when virulent strains are uniquely capable of overcoming pre-existing immunity. Consistent with this possibility, we found that the within-host advantage of strain virulence, as measured by quantitative pathogen load, was mediated by the degree of prior exposure. Specifically, the largest pathogen load advantage of virulence was seen in hosts with prior exposure to repeated low doses of MG (Fig. 2). While virulent strains infecting hosts that were previously primed with repeated low-levels of MG [6(10^1^)] were able to replicate to levels equivalent to those in immunologically naïve hosts (Fig. 2), the two less virulent strains were effectively “shut out” by any degree of pathogen priming used in this study. Thus, our results suggest that the same priming exposure levels can act in either a qualitative (“all-or-nothing”) or dose-dependent fashion depending on the virulence of the challenge strain. Although our experimental design did not allow us to robustly exclude contributions of strain homology, or other strain-specific traits in altering host responses to secondary infection, past work on this system^11,21,22^ and supplementary analyses (Table S7) indicate that effects of homology do not alter the detected effects of virulence and priming exposure level on quantitative pathogen load. Further, our results are consistent with prior work on this system that used larger numbers of strains of varying virulence^11^.

Our recent work in this system demonstrated that incomplete host immunity produced by prior exposure strongly favors the evolution of more virulent pathogens^11^. However, the doses used to mimic prior exposure in our previous study were uniformly high, and priming exposures varied in strain virulence but not exposure level. Our results here suggest that the effects of incomplete immunity on virulence evolution generated by high exposure doses in our prior work^11^ may be even stronger in natural populations, where exposure levels are heterogeneous. Although the current study only varied the dose associated with priming exposure, future work should additionally examine the effect of the secondary challenge dose on within-host load and disease severity. Selection for virulent pathogens may be even stronger in cases where both the priming and challenge doses are low, a scenario that may better reflect exposure levels in natural populations. Finally, our results as compared to prior work^11^ suggest that the timing of secondary challenge relative to priming exposure influences the strength of incomplete host immunity in this system. While the most common interval between repeated exposures in wild house finches not known, in this study the temporal window between primary and secondary inoculations was designed to replicate reinfections that may occur within an epidemic cycle, such as the one experienced by free-living finches between the months of September and November^28^. The stronger protection against high-dose reinfection detected in this study versus our prior work^11^, where reinfections occurred after a longer temporal window (~4.5 months), suggests an effect of waning immunity in this system^42^. Waning immunity is an important consideration when assessing population-level selection pressure on a pathogen^12,43^, as a completely protected individual may become incompletely protected from one epidemic season to another.

Intriguingly, we found that strain virulence and prior exposure level not only altered mean pathogen loads, but also altered the degree of variation in pathogen loads (Fig. 3). The most virulent strain generally showed the highest degree of variability in pathogen load regardless of host immune background, but this variation was most pronounced in the groups with previous low-level exposure. This suggests that, although the most virulent strain harbored the highest overall pathogen loads, it does not do equally well in all hosts, particularly if hosts experienced very low-level prior exposures that may be more likely to generate highly variable degrees of immune protection. Heterogeneity in susceptibility in a host population may then lead to a trade-off for virulent pathogens, if more virulent strains have higher mean transmission but also more variation in transmission^44^. Future work should examine the immunological mechanisms associated with protection from secondary infection, as these mechanisms may shed light on important sources of heterogeneity in susceptibility in this system.

In addition to amplifying the quantitative pathogen load advantage of virulence, incomplete immunity formed from prior low-level exposure also reduced the costs of virulence to the pathogen, which we measured as disease severity following secondary challenge (Fig. 4). Under the trade-off model, directly-transmitted pathogens suffer costs of virulence in the form of increased morbidity and mortality, which are expected to reduce contact rates and infectious periods, thus reducing opportunities for transmission. Here we found that the most virulent strain was able to maintain a quantitative pathogen load advantage in incompletely immune hosts, without inducing proportional levels of disease severity as it does in hosts with no prior exposure (‘None (sham)’ group; Fig. 4). While prior exposure significantly reduced the costs of virulence for all strains (Fig. 4), strains of high virulence should benefit the most from this reduction because the costs of virulence are highest for these strains in the absence of any pre-existing immunity. Our metric of disease severity (clinical signs of conjunctivitis) has been found to positively correlate with reductions in anti-predator behavior^30^, suggesting that the reduction in disease severity observed in incompletely immune hosts might allow increased pathogen transmission and persistence of virulent strains in wild populations that contain hosts with pre-existing immunity from prior exposure. Future work should integrate the effects of pre-existing immunity on both disease severity and quantitative pathogen load to determine how both together determine optimal pathogen virulence in hosts with varying degrees of prior immunity. One limitation of our study is that we only quantified proxy for within-host fitness of pathogen strains, while the ultimate metric of pathogen fitness is between-host transmission. Although we did not explicitly test how transmission potential changes with a host’s pre-existing immunity, quantitative pathogen loads appear to have important implications for transmission in this system. Previous work showed that as conjunctival pathogen loads increase in house finches, so does pathogen deposition onto feeders^41^, an important transmission source in the wild^26^. Thus, differences in quantitative pathogen loads likely have relevant consequences for transmission in this system. However, given that prior work examined how pathogen loads predict transmission potential in immunologically-naïve hosts alone, future work should quantify between-host fitness when determining the role of prior exposure in shaping the disease dynamics of this songbird-bacterium system.

Together, our results indicate that prior exposure level may be an important mediator of within-host pathogen dynamics and selection for higher virulence, particularly where low-level exposures produce incomplete immunity. Incompletely immune house finches appear to provide a within-host environment for MG that both favors virulence and reduces the costs of maintaining virulence, enhancing the fitness potential of virulent pathogens in populations where hosts have repeated infections. This is particularly relevant in systems, like house finches, where there is significant variation in exposure level and where reinfections are possible. There is increasing evidence that exposure level may be an important mediator of host heterogeneity in natural populations [e.g.^19,45^], where humans and free-living wildlife are expected to vary in both their distribution in and use of a landscape, altering the potential for pathogen exposure [e.g.^46,47^]. Similarly, there is evidence that reinfections, either due to waning immunity [e.g.^48^] or imperfect immunity [e.g.^13^], are common across host-pathogen systems. Variation in exposure history is thus an important consideration for anticipating and understanding the evolution of more harmful pathogens in natural populations where reinfections occur.

## Supplementary information

Supplementary Material.

## Data Availability

The datasets generated and analyzed during the current study are available in the Dryad digital repository, https://datadryad.org/review?doi=doi:10.5061/dryad.rb23s12.
