# Supplementary material for: Host exposure history modulates the within-host advantage of virulence in a songbird-bacterium system
Source: Sci Rep. 2019 Dec 30;9:20348. doi: 10.1038/s41598-019-56540-6 (PMC6937340; doi:10.1038/s41598-019-56540-6)
Supplement: Supplementary file 1 — Supplementary Material. [file 41598_2019_56540_MOESM1_ESM.docx]

**Host exposure history modulates the within-host advantage of virulence in a songbird-bacterium system**

Leon AE, Fleming-Davies AE and Hawley DM

**SUPPLEMENTARY MATERIAL**

**Experimental Design**

***Table S1****.* Experimental treatment groups are shown by priming and secondary exposure treatments. Individuals received one of five priming exposure treatments which varied by dose and total number of inoculations. After recovering from priming exposures, each individual received a single high-dose secondary inoculation with one of three strains which varied in virulence.

| **Table S1. Priming and Secondary Exposure Groups** | | **Priming exposure (CCU/mL)** | | | | |
| --- | --- | --- | --- | --- | --- | --- |
|  |  | **6(10^1^)** | **1(10^2^)** | **6(10^2^)** | **1(10^6^)** | **Sham** |
| **Secondary**  **Exposure** | **Lower virulence** | N=7 | N = 7 | N = 7 | N = 7 | N = 7 |
|  | **Equal virulence**  **(homologous)** | N = 8 | N = 6 | N = 6 | N = 6 | N= 6 |
|  | **Higher virulence** | N=7 | N = 7 | N = 7 | N = 7 | N = 7 |
|  | **Sham** |  |  |  |  | N = 2 |

**Inoculations**

All stock inocula were grown using Frey’s broth media with 15% swine serum (FMS) and stored at -80°C prior to use. The day of inoculations, inocula were thawed and diluted to approximate dosages immediately prior to use. All inoculations (MG or sham) consisted of a total volume of 70uL of inoculum distributed equally into both conjunctivae of each bird using a 100uL micropipette. On days where not all individuals received an inoculation, a random subset of individuals across treatments were given a sham inoculation of media alone to control for the stress of handling and the effect of media alone in the ocular tissue. There was no statistical effect of this sham treatment on disease or infection outcomes as compared to animals that did not receive this treatment (data not shown).

**Quantitative Pathogen Load**

Conjunctival sacs were swabbed for 5 seconds using sterile swabs dipped in tryptose phosphate broth (TPB). Swabs from both eyes were eluted in a single tube containing 300uL of TPB. Samples were kept on ice until they could be frozen at -20°C. Samples remained frozen until thawed for DNA extraction. DNA was extracted using Qiagen DNeasy 96 Blood and Tissue kits (Qiagen, Valencia, CA) and qPCR was performed using primers and a probe that target the Mgc2 gene of MG [33]. A standard curve of 2.98 x 10^1^ to 2.98 x 10^8^ copy numbers was produced using a plasmid containing a 303 bp Mgc2 insert [33].The following cycling parameters were used: 95°C for 3 minute then 40 cycles of 95°C for 3 seconds followed by 60°C for 30 seconds. Mgc2 copy numbers were log10 transformed prior to statistical analysis. Cycling was performed using Bio-Rad C1000 CFX96 Real-time System (Hercules, CA).

**Final sample sizes**

Treatments [6(10^1^)], [6(10^2^)] and Sham all lost one individual due to mortality during primary infection, while groups [6(10^1^)], [1(10^2^)], [1(10^6^)] and Sham all lost one individual during secondary infection. Additionally, one individual from group [1(10^6^)] was excluded from secondary infection due to chronic infection (persistence of eye score and pathogen load) from primary exposure.

**Primary Infection Methods and Analyses**

Animals were sampled for pathogen load and the severity of clinical signs on post-priming inoculation days 4, 6, 13, 20, and 27. However, only a subset of pathogen load samples that captured the relevant temporal dynamics of infection were analyzed (days 6 and 20 post-priming exposure).

Because we were interested in the differences in infection and disease outcomes across pathogen treatments, sham control animals were excluded from primary infection analyses. All sham control animals had zero values for both pathogen load and eye score during primary infection, with the exception of one individual at one-time point. This individual displayed a pathogen load of 2.26 log_10_ copies day 6 post-inoculation, but had a pathogen load of zero for all other time points and at no point displayed any clinical signs of disease. This pathogen load is likely due to contamination of the sample at some point in sampling or DNA extraction, as previously recorded for this highly sensitive assay in Leon & Hawley [17].To account for potential contamination, we included a total of 32 extraction controls (blank samples that underwent the extraction and PCR process alongside actual samples) and 15 environmental controls (swabs waved in the air at the time of sampling and then eluted in buffer and treated like all other samples) dispersed throughout our samples of interest. These samples showed a much lower rate of background contamination than previously recorded [17] with 4/47 (*8.5%) coming up qPCR positive at low-levels. In addition to assay controls, we also included 2 MG-negative individuals in the experiment as controls for both primary and secondary infection periods (Supp. Table 1), which at no point were found to be qPCR positive. These animals were not included in analyses.

*Pathogen Load Analysis*

Pathogen loads from primary timepoints days 6 and 20 post-inoculation for each individual were averaged before analysis. For primary infection, differences in pathogen load were analyzed using a generalized linear model with a Gamma distribution with an inverse link function. Consistent with previous work [17], Gamma distribution was selected for this model due to a non-normal distribution of the residuals using a linear model, and because our response variable, pathogen load, is a continuous positive variable. Priming treatment was our fixed effect of interest.

*Disease Severity Analysis*

Eye scores, our measure of disease severity, were averaged across primary infection for each individual before analysis. Post-priming inoculation sampling dates were corrected based on an individual’s first date of inoculation, which did not coincide across groups due variation in the total number of inoculations individuals received (Fig. 1). We did this in order to capture equivalent temporal windows of infection for all groups. Thus, groups [6(10^1^)] and [6(10^2^)] were sampled for clinical signs on post-priming inoculation days 6, 14, and 23, while groups [1(10^2^)] and [1(10^6^)] were sampled for clinical signs on post-priming inoculation days 6, 13 and 20. We were able to correct for this discrepancy for eye scores due to the high resolution of samples collected. Primary infection eye score responses were analyzed using a generalized linear model assuming a Poisson distribution and log link function, with priming treatment as our fixed effect.

**Primary Infection Results**

Consistent with previous results [22], quantitative pathogen load during primary infection was significantly altered by exposure level (generalized linear model assuming a Gamma distribution; exposure level: LR = 19.98, df = 3, P <0.001; Supp. Fig. 1, Supp. Tab. 1). Overall, pathogen loads in response to repeated exposures appeared more variable as compared to single exposure groups (Supp. Fig. 1). Disease severity during primary infection, measured via the extent of clinical signs, also significantly increased with priming exposure level (glm assuming a Poisson distribution; exposure level: LR = 19.56, df = 3, P <0.001; Fig. 2, Supp. Tab. 2), consistent with previous findings [17].

***Table S2.*** Parameter estimates for a generalized linear model assuming a Gamma distribution (response on the inverse scale) used to assess how quantitative pathogen loads were altered by priming exposure level in house finches experimentally inoculated with *Mycoplasma gallisepticum*.

|  | Estimate | Std. Error | z value | Pr(>\|z\|) |
| --- | --- | --- | --- | --- |
| Exposure level - 6(10^1^) | 0.6373 | 0.0659 | 9.68 | 6.8e-15 |
| Exposure level - 1(10^2^) | -0.1414 | 0.0842 | -1.68 | 0.0974 |
| Exposure level - 6(10^2^) | 0.1581 | 0.1087 | 1.45 | 0.1499 |
| Exposure level - 1(10^6^) | -0.2152 | 0.0796 | -2.70 | 0.0085 |

***Table S3*.** Parameter estimates for a generalized linear model assuming a Poisson distribution (response on the log scale) used to assess how visible eye scores were altered by priming exposure level in house finches experimentally inoculated with *Mycoplasma gallisepticum*.

|  | Estimate | Std. Error | z value | Pr(>\|z\|) |
| --- | --- | --- | --- | --- |
| Exposure level - 6(10^1^) | -1.946 | 0.577 | -3.37 | 0.00075 |
| Exposure level - 1(10^2^) | 0.560 | 0.730 | 0.77 | 0.44351 |
| Exposure level - 6(10^2^) | 0.611 | 0.730 | 0.84 | 0.40286 |
| Exposure level - 1(10^6^) | 1.946 | 0.619 | 3.14 | 0.00167 |

**
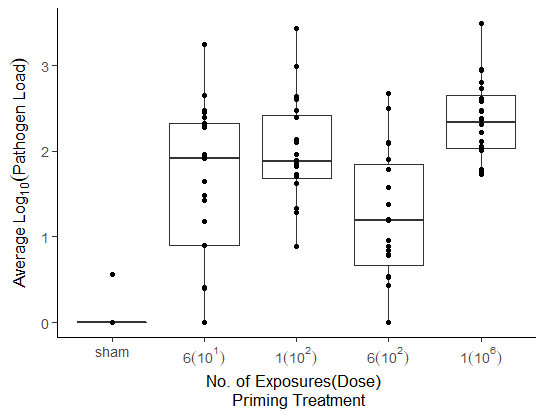
**

**Figure S1.** Quantitative pathogen loads of Mycoplasma gallisepticum in the conjunctiva of house finches immediately following priming exposures. Conjunctival pathogen load generally increased with priming exposure level of Mycoplasma gallisepticum. Higher variability was seen in groups with repeated exposures. Average pathogen load includes conjunctival loads averaged across primary infection sampling time-points. Each point represents an individual.

**
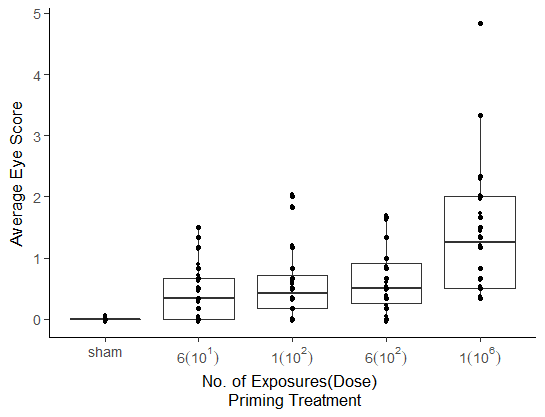
**

**Figure S2**. Disease severity in house finches immediately following priming exposures of Mycoplasma gallisepticum. Disease severity increased with priming dose and number of exposures of Mycoplasma gallisepticum. Eye scores were measured on a scale of 0-3 for each eye and summed across eyes. Points are average eye scores per individual, with scores for both eyes summed within primary infection sampling time-points and then averaged across time points.

***Table S4*.** Parameter estimates for a linear mixed model used to assess how quantitative pathogen load was altered by priming exposure level, secondary challenge strain and the interaction of priming exposure level and secondary strain in house finches experimentally inoculated with *Mycoplasma gallisepticum.* Individual ID was used as the random effect to account for repeated measures (individuals: 95, observations: 284).

|  | Estimate | Std. Error | t value |
| --- | --- | --- | --- |
| Exposure level - Sham | 2.3239 | 0.4350 | 5.34 |
| Exposure level - 6(10^1^) | -1.6606 | 0.6152 | -2.70 |
| Exposure level - 1(10^2^) | -2.0372 | 0.5928 | -3.44 |
| Exposure level - 6(10^2^) | -1.6206 | 0.6152 | -2.63 |
| Exposure level - 1(10^6^) | -1.2453 | 0.5928 | -2.10 |
| Secondary Strain - VA94 | 1.6861 | 0.6152 | 2.74 |
| Secondary Strain - NC06 | 2.4690 | 0.5928 | 4.16 |
| Exposure level - 6(10^1^):  Secondary Strain - VA94 | -1.5590 | 0.8543 | -1.82 |
| Exposure level - 1(10^2^):  Secondary Strain - VA94 | -1.5993 | 0.8576 | -1.86 |
| Exposure level - 6(10^2^):  Secondary Strain - VA94 | -2.0400 | 0.8700 | -2.34 |
| Exposure level - 1(10^6^):  Secondary Strain - VA94 | -1.9767 | 0.8762 | -2.26 |
| Exposure level - 6(10^1^):  Secondary Strain - NC06 | 0.0887 | 0.8383 | 0.11 |
| Exposure level - 1(10^2^):  Secondary Strain - NC06 | -0.5579 | 0.8383 | -0.67 |
| Exposure level - 6(10^2^):  Secondary Strain - NC06 | -1.8190 | 0.8383 | -2.17 |
| Exposure level - 1(10^6^):  Secondary Strain - NC06 | -2.4253 | 0.8383 | -2.89 |

***Table S5.*** Post-hoc pair-wise comparisons for a linear mixed model (Table S4) used to assess how quantitative pathogen load was altered by priming exposure level, secondary challenge strain and the interaction of priming exposure level and secondary strain in house finches experimentally inoculated with *Mycoplasma gallisepticum.* Pair-wise comparisons were done across priming exposure levels within each secondary challenge strain.

|  | Estimate | Std. Error | df | t ratio | p value |
| --- | --- | --- | --- | --- | --- |
| Secondary Strain – CA06 |  |  |  |  |  |
| Sham - 6(10^1^) | 1.6606 | 0.615 | 79.7 | 2.70 | 0.0816 |
| Sham - 1(10^2^) | 2.0372 | 0.593 | 79.7 | 3.44 | 0.0093 |
| Sham - 6(10^2^) | 1.6206 | 0.615 | 79.7 | 2.63 | 0.0968 |
| Sham - 1(10^6^) | 1.2453 | 0.593 | 79.7 | 2.10 | 0.3270 |
| 6(10^1^) - 1(10^2^) | 0.3767 | 0.593 | 79.7 | 0.635 | 0.9994 |
| 6(10^1^) - 6(10^2^) | -0.0400 | 0.615 | 79.7 | -0.065 | 1.0000 |
| 6(10^1^) - 1(10^6^) | -0.4152 | 0.593 | 79.7 | -0.700 | 0.9987 |
| 1(10^2^) - 6(10^2^) | -0.4167 | 0.593 | 79.7 | -0.703 | 0.9987 |
| 1(10^2^) - 1(10^6^) | -0.7919 | 0.570 | 79.7 | -1.390 | 0.8416 |
| 6(10^2^) - 1(10^6^) | -0.3752 | 0.593 | 79.7 | -0.633 | 0.9995 |
| Secondary Strain – VA94 |  |  |  |  |  |
| Sham - 6(10^1^) | 3.2195 | 0.593 | 79.7 | 5.431 | <.0001 |
| Sham - 1(10^2^) | 3.6365 | 0.620 | 81.8 | 5.867 | <.0001 |
| Sham - 6(10^2^) | 3.6605 | 0.615 | 79.7 | 5.950 | <.0001 |
| Sham - 1(10^6^) | 3.2220 | 0.645 | 79.7 | 4.994 | <.0001 |
| 6(10^1^) - 1(10^2^) | 0.4169 | 0.598 | 81.9 | 0.698 | 0.9987 |
| 6(10^1^) - 6(10^2^) | 0.4410 | 0.893 | 79.7 | 0.744 | 0.9979 |
| 6(10^1^) - 1(10^6^) | 0.0025 | 0.624 | 79.7 | 0.004 | 1.0000 |
| 1(10^2^) - 6(10^2^) | 0.0241 | 0.620 | 81.8 | 0.039 | 1.0000 |
| 1(10^2^) - 1(10^6^) | -0.4145 | 0.650 | 81.6 | -0.638 | 0.9994 |
| 6(10^2^) - 1(10^6^) | -0.4386 | 0.645 | 79.7 | -0.680 | 0.9990 |
| Secondary Strain – NC06 |  |  |  |  |  |
| Sham - 6(10^1^) | 1.5719 | 0.570 | 79.7 | 2.760 | 0.0694 |
| Sham - 1(10^2^) | 2.5951 | 0.593 | 79.7 | 4.378 | 0.0004 |
| Sham - 6(10^2^) | 3.4395 | 0.570 | 79.7 | 6.039 | <.0001 |
| Sham - 1(10^6^) | 3.6706 | 0.593 | 79.7 | 6.192 | <.0001 |
| 6(10^1^) - 1(10^2^) | 1.0231 | 0.593 | 79.7 | 1.726 | 0.6029 |
| 6(10^1^) - 6(10^2^) | 1.8676 | 0.570 | 79.7 | 3.279 | 0.0153 |
| 6(10^1^) - 1(10^6^) | 2.0987 | 0.593 | 79.7 | 3.540 | 0.0067 |
| 1(10^2^) - 6(10^2^) | 0.8444 | 0.593 | 79.7 | 1.425 | 0.8213 |
| 1(10^2^) - 1(10^6^) | 1.0756 | 0.615 | 79.7 | 1.748 | 0.5853 |
| 6(10^2^) - 1(10^6^) | 0.2311 | 0.593 | 79.7 | 0.90 | 1.0000 |

***Table S6*.** Parameter estimates for a reduced linear mixed model (sham group excluded) to assess how quantitative pathogen load was altered by priming exposure level, secondary challenge strain and the interaction of priming exposure level and secondary strain in house finches experimentally inoculated with *Mycoplasma gallisepticum.* Individual ID was used as the random effect to account for repeated measures (individuals: 76, observations: 227).

|  | Estimate | Std. Error | t value |
| --- | --- | --- | --- |
| Exposure level - 6(10^1^) | 0.66333 | 0.44709 | 1.484 |
| Exposure level - 1(10^2^) | -0.37667 | 0.60928 | -0.618 |
| Exposure level - 6(10^2^) | 0.04000 | 0.63228 | 0.063 |
| Exposure level - 1(10^6^) | 0.41524 | 0.60928 | 0.682 |
| Secondary Strain - VA94 | 0.12714 | 0.60928 | 0.209 |
| Secondary Strain - NC06 | 2.55762 | 0.60928 | 4.198 |
| Exposure level - 1(10^2^):  Secondary Strain - VA94 | -0.03997 | 0.86419 | -0.046 |
| Exposure level - 6(10^2^):  Secondary Strain - VA94 | -0.48103 | 0.87806 | -0.548 |
| Exposure level - 1(10^6^):  Secondary Strain - VA94 | -0.41771 | 0.88454 | -0.472 |
| Exposure level - 1(10^2^):  Secondary Strain - NC06 | -0.64651 | 0.86165 | -0.750 |
| Exposure level - 6(10^2^):  Secondary Strain - NC06 | -1.90762 | 0.86165 | -2.214 |
| Exposure level - 1(10^6^):  Secondary Strain - NC06 | -2.51397 | 0.86165 | -2.918 |

***Table S7*.** Parameter estimates for a linear mixed model used to assess how quantitative pathogen load was altered by priming exposure level, secondary challenge strain, the interaction of priming exposure level and secondary strain, and the effect of strain homology in house finches experimentally inoculated with *Mycoplasma gallisepticum.* Here virulence is treated as a quantitative variable and strain homology is categorical (yes or no). Individual ID was used as the random effect to account for repeated measures (individuals: 95, observations: 284).

|  | Estimate | Std. Error | t value |
| --- | --- | --- | --- |
| Exposure level - Sham | 1.5289 | 0.6375 | 2.386 |
| Exposure level - 6(10^1^) | -2.1563 | 0.8956 | -2.401 |
| Exposure level - 1(10^2^) | -2.1147 | 0.8745 | -2.418 |
| Exposure level - 6(10^2^) | -1.116 | 0.8968 | -1.245 |
| Exposure level - 1(10^6^) | -0.4067 | 0.8752 | -0.465 |
| Secondary Strain Virulence | 0.7583 | 0.1808 | 4.193 |
| Homology (yes) | -0.6462 | 0.2392 | -2.701 |
| Exposure level - 6(10^1^):  Secondary Strain Virulence | 0.00382 | 0.2558 | 0.015 |
| Exposure level - 1(10^2^):  Secondary Strain Virulence | -0.1962 | 0.2549 | -0.769 |
| Exposure level - 6(10^2^):  Secondary Strain Virulence | -0.5631 | 0.2558 | -2.202 |
| Exposure level - 1(10^6^):  Secondary Strain Virulence | -0.7328 | 0.2550 | -2.873 |

***Table S8*.** Parameter estimates for a linear mixed model used to assess how disease severity, measured via visible eye lesions, was altered by priming exposure level, secondary challenge strain and the interaction of the two in house finches experimentally inoculated with *Mycoplasma gallisepticum.* Individual ID was used as the random effect to account for repeated measures (individuals: 95, observations: 284).

|  | Estimate | Std. Error | t value |
| --- | --- | --- | --- |
| Exposure level - Sham | 0.1221 | 0.0946 | 1.29 |
| Exposure level - 6(10^1^) | -0.0610 | 0.1338 | -0.46 |
| Exposure level - 1(10^2^) | -0.1221 | 0.1290 | -0.95 |
| Exposure level - 6(10^2^) | -0.1221 | 0.1338 | -0.91 |
| Exposure level - 1(10^6^) | -0.1221 | 0.1290 | -0.95 |
| Secondary Strain - VA94 | 0.5000 | 0.1338 | 3.74 |
| Secondary Strain - NC06 | 0.8791 | 0.1290 | 6.82 |
| Exposure level - 6(10^1^):  Secondary Strain - VA94 | -0.5087 | 0.1858 | -2.74 |
| Exposure level - 1(10^2^):  Secondary Strain - VA94 | -0.4767 | 0.1867 | -2.55 |
| Exposure level - 6(10^2^):  Secondary Strain - VA94 | -0.4389 | 0.1892 | -2.32 |
| Exposure level - 1(10^6^):  Secondary Strain - VA94 | -0.5000 | 0.1906 | -2.62 |
| Exposure level - 6(10^1^):  Secondary Strain - NC06 | -0.5005 | 0.1824 | -2.74 |
| Exposure level - 1(10^2^):  Secondary Strain - NC06 | -0.6640 | 0.1824 | -3.64 |
| Exposure level - 6(10^2^):  Secondary Strain - NC06 | -0.7744 | 0.1824 | -4.25 |
| Exposure level - 1(10^6^):  Secondary Strain - NC06 | -0.7061 | 0.1824 | -3.87 |
